# Incarceration of the Gravid Uterus: Proposal for a Shared Definition. Comment on Tachibana et al. Incarcerated Gravid Uterus: Spontaneous Resolution Is Not Rare. *Diagnostics* 2021, *11*, 1544

**DOI:** 10.3390/diagnostics12010021

**Published:** 2021-12-23

**Authors:** Michele Orsi, Edgardo Somigliana

**Affiliations:** 1 Maternal-Infant Department, A.S.S.T. Rhodense, Garbagnate Milanese, Via Carlo Forlanini, 95, 20024 Milan, Italy; 2 Department of Woman, New-Born and Child, Fondazione IRCCS Ca’ Granda Ospedale Maggiore Policlinico, Mangiagalli Centre, Via della Commenda, 12, 20122 Milan, Italy; dadosomigliana@yahoo.it; 3 Department of Clinical Sciences and Community Health, Università degli Studi di Milano, Via Festa del Perdono, 7, 20122 Milan, Italy

We read with great interest the paper entitled “Incarcerated gravid uterus: spontaneous resolution is not rare” by Tachibana et al. [1]. The report is relevant for at least three aspects. First, the case series is the largest reported for this infrequent clinical entity. Second, it underlines that a “wait and see” approach must be the first choice in the absence of severe complications. Third, the hospital-based framework and the 10-year study period allowed calculation of the incidence, which revealed higher frequency compared with previous reports [2]. However, the definition adopted could lead to underestimation of the phenomenon. The choice of 16 weeks of gestation as inclusion threshold is restrictive, since numerous reports have described incarceration of the retroverted gravid uterus associated with severe symptoms at a lower gestational age. Threatened miscarriage, acute urinary retention requiring bladder catheterization, manual repositioning of the uterus and hospitalization have even been reported in the first trimester of pregnancy [3,4].

As appropriately stated by the authors, a shared definition of uterine incarceration should include symptomatic cases, as asymptomatic cases could be considered variations of the normal anatomy and spontaneous repositioning has been reported even in the third trimester of pregnancy [1,5,6,7]. However, insidious cases with asymptomatic and unrecognized incarceration at term (i.e., Case 14) could experience sudden and severe obstetric complications, such as obstructed labour, cervical or vaginal incision at caesarean section, bladder injury and massive haemorrhage [1,8,9,10,11].

On these bases, we believe that the definition should include both symptomatic cases at any gestational age and asymptomatic cases at birth potentially at risk of catastrophic complications. This option would take into account early-onset cases, as well as the possibility of spontaneous repositioning at any gestation age.
